# The gamma-glutamyltransferase-to-estimated glomerular filtration rate composite index is associated with arterial stiffness: a cross-sectional secondary analysis of a health check-up population

**DOI:** 10.3389/fendo.2026.1930792

**Published:** 2026-08-28

**Authors:** Tengfei Gu, Guanghui Li, Gen Li, Pan Gao, Yang Su, Xiaoyang Li, Ziniu Zhao

**Affiliations:** Department of Cardiac Surgery, Fuwai Central China Cardiovascular Hospital, Henan Cardiovascular Disease Center (Central China Subcenter of National Center for Cardiovascular Diseases), Central China Fuwai Hospital of Zhengzhou University, Henan Provincial Cardiovascular Hospital, Zhengzhou, China

**Keywords:** arterial stiffness, brachial-ankle pulse wave velocity, cardiometabolic risk, composite index, estimated glomerular filtration rate, gamma-glutamyltransferase, oxidative stress

## Abstract

**Background:**

Elevated gamma-glutamyltransferase (GGT), a marker of oxidative stress and cardiometabolic burden, and reduced estimated glomerular filtration rate (eGFR) are each linked to arterial stiffness, but they are usually examined separately. This study evaluated whether a single index integrating both—the GGT–eGFR composite index—is associated with brachial-ankle pulse wave velocity (baPWV).

**Methods:**

This was a cross-sectional analysis of 893 adults from a Japanese health check-up program. The composite index was calculated as ln(GGT) × 100/eGFR. Its association with baPWV (continuous) and with elevated baPWV (≥1400 and ≥1600 cm/s) was assessed using multivariable linear and logistic regression, restricted cubic splines, joint GGT×eGFR stratification, subgroup analyses, and sensitivity analyses. Models containing the composite index were also compared with models containing ln(GGT), eGFR, or both components.

**Results:**

baPWV increased across index quartiles (1295 to 1531 cm/s). In the fully adjusted model, each 1-SD increment in the index was associated with a 20.46 cm/s higher baPWV (95% CI 5.64–35.28; P = 0.007); the highest versus lowest quartile difference was 74.77 cm/s (95% CI 33.96–115.57; P<0.001). The index was also associated with baPWV ≥1400 cm/s (OR 1.32, 95% CI 1.06–1.64) and ≥1600 cm/s (OR 1.36, 95% CI 1.04–1.79). Direct model comparisons showed a modest improvement in model fit for continuous baPWV after inclusion of the composite index, while categorical model performance was broadly comparable across models. Restricted cubic spline analysis showed an overall association with baPWV (P for overall = 0.011), with no statistically significant evidence of departure from linearity (P for non-linearity = 0.192). The association was consistent across sensitivity analyses.

**Conclusion:**

In this cross-sectional secondary analysis, a higher exploratory GGT–eGFR-derived index remained associated with higher baPWV after multivariable adjustment. Direct comparison with ln(GGT) and eGFR showed only modest differences in model fit, and prospective external validation is required before clinical application can be considered.

## 1 Introduction

Arterial stiffening is one of the earliest measurable steps in vascular ageing. As the elastic arteries lose compliance, pulsatile load on the heart and the microcirculation rises, and this precedes the clinical manifestations of cardiovascular disease by years (1). Brachial-ankle pulse wave velocity (baPWV) is a simple, automated, and reproducible measure of systemic arterial stiffness that is widely used in East Asian health examinations, and higher values predict cardiovascular events and mortality independently of conventional risk factors (2–7). Identifying inexpensive markers that track this subclinical process is therefore useful for early risk stratification.

Two routinely measured laboratory variables have each been tied to arterial stiffness through partly overlapping pathways. Serum gamma-glutamyltransferase (GGT), long regarded as a liver enzyme, is now recognized as a marker of systemic oxidative stress and cardiometabolic burden: it depletes extracellular glutathione, generates reactive oxygen species that oxidize low-density lipoprotein, and is found catalytically active within atherosclerotic plaques (8–13). Higher GGT is associated with metabolic syndrome, non-alcoholic fatty liver disease, incident diabetes, and cardiovascular mortality (14–17), and several studies have linked it to increased baPWV (18–20). Reduced estimated glomerular filtration rate (eGFR), in turn, marks impaired renal function and is accompanied by fluid overload, mineral dysregulation, chronic inflammation, and accelerated vascular calcification; even modest declines in eGFR are associated with stiffer arteries and worse cardiovascular outcomes (21–25).

Although GGT and eGFR reflect different physiological domains, they converge on several pathways relevant to arterial stiffening. Higher GGT is associated with increased oxidative stress and cardiometabolic burden, which can impair endothelial function and promote vascular remodeling, whereas reduced eGFR is accompanied by chronic inflammation, disturbances in mineral metabolism, and vascular calcification. These processes may act in parallel on the arterial wall and contribute to loss of arterial compliance. A measure that increases with both higher GGT and lower eGFR may therefore summarize two complementary dimensions of systemic vascular burden within a single term. Because the adverse directions of the two markers are opposite, we constructed the index so that higher GGT increases its value whereas lower eGFR also increases it. This construction was intended as an exploratory summary measure and does not imply a biological interaction or synergistic effect between GGT and eGFR. Several composite measures derived from routine laboratory variables have been examined in relation to arterial stiffness in this and similar populations (26–30), but GGT and eGFR have not previously been combined in this context.

To address this gap, a GGT–eGFR composite index was constructed, defined so that a higher value reflects both greater GGT and lower eGFR, and its association with baPWV was examined in a Japanese health check-up population. The relationship was characterized across the full range of the index, the individual contributions of GGT and eGFR and their statistical interaction were examined, and the consistency of the association was tested across clinically relevant subgroups. Models containing the composite index were also compared directly with models containing ln(GGT), eGFR, or both components to examine whether the derived index provided additional statistical information.

## 2 Materials and methods

### 2.1 Study design, data source, and ethics

This was a cross-sectional secondary analysis of a publicly available dataset. The original study was conducted at the Medical Health Check-up Center of Murakami Memorial Hospital (Gifu, Japan) between March 2004 and December 2012, in which participants underwent a health examination that included baPWV measurement and abdominal ultrasonography; the de-identified data were deposited by Fukuda et al. in the Dryad Digital Repository (doi:10.5061/dryad.m484p) (31). The original study was approved by the institutional ethics committee and all participants provided written informed consent. Because the present analysis used fully anonymized, publicly available data, additional ethical approval and informed consent were not required. The work followed the STROBE recommendations for cross-sectional studies.

**Figure 1:**
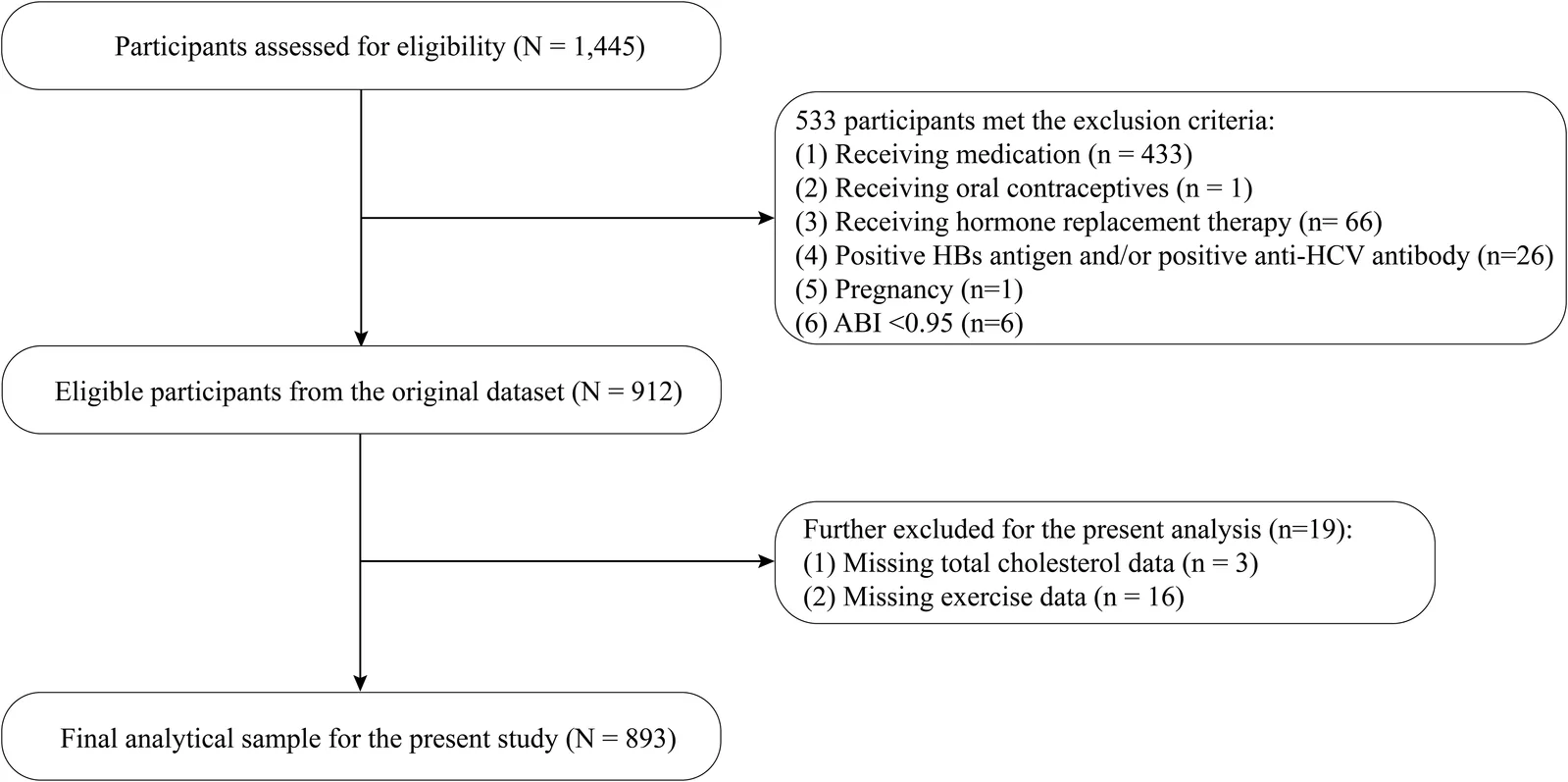
Flow diagram of participant selection. Of 1,445 individuals assessed for eligibility, 533 met exclusion criteria and 19 were further excluded for missing total cholesterol or exercise data, leaving 893 for analysis.

Of 1,445 individuals assessed for eligibility, 533 were excluded because they were taking medication (n = 433) or oral contraceptives (n = 1), were receiving hormone replacement therapy (n = 66), were positive for hepatitis B surface antigen and/or anti-hepatitis C antibody (n = 26), were pregnant (n = 1), or had an ankle-brachial index (ABI) < 0.95 (n = 6), leaving 912 eligible participants in the public dataset. For the present secondary analysis, the analytical dataset was reconstructed *de novo* from these 912 participants, and 19 were further excluded for missing total cholesterol (n = 3) or exercise (n = 16) data, yielding a final analytical sample of 893 (**Figure 1**). Thirteen participants with missing alcohol-consumption data were not excluded; instead, missing alcohol status was retained as a separate category, so that all 893 participants were included in every regression model. Participants were divided into quartiles (Q1–Q4) of the GGT–eGFR composite index.

### 2.2 Measurements and definitions

baPWV was measured with an automated waveform analyzer, and ABI was recorded concurrently. ABI values were checked for apparent decimal-place formatting errors. Thirty-five values recorded as 100 or greater were divided by 1,000, and one value recorded as 13.05 was divided by 10. The corrected ABI values were used in the primary analyses. A sensitivity analysis was conducted after excluding all 36 participants whose ABI values required correction. Blood was sampled after an overnight fast for GGT, aspartate and alanine aminotransferases, fasting plasma glucose, uric acid, total cholesterol, triglycerides, high- and low-density lipoprotein cholesterol, and serum creatinine. eGFR was estimated using the Japanese creatinine-based equation: 194 × serum creatinine^−1.094^ × age^−0.287^ × 0.739 for women (32). Fatty liver was diagnosed by abdominal ultrasonography according to standardized criteria (33). Among women, menopausal status was obtained by questionnaire, with postmenopause defined as beginning 1 year after cessation of menses, consistent with the source study. Body mass index (BMI) was weight divided by height squared. Smoking status was reconstructed from the current-smoking and former-smoking variables into three mutually exclusive categories. Participants were classified as current smokers when the current-smoking indicator was positive, as former smokers when the current-smoking indicator was negative and the former-smoking indicator was positive, and as never smokers when both indicators were negative. Exercise habit and alcohol consumption were obtained using a standardized questionnaire. Weekly alcohol intake was estimated in grams and categorized as none/minimal (<40 g/week), light (40–140 g/week), moderate (140–280 g/week), or heavy (>280 g/week), consistent with the source study (31). Regular exercise was defined as participation in sports or recreational activity at least once per week.

### 2.3 GGT–eGFR composite index and outcomes

For the present analysis, the GGT–eGFR composite index was defined as ln(GGT) × 100/eGFR. GGT was log-transformed because of its right-skewed distribution. This formulation places higher GGT and lower eGFR in the same direction, with both resulting in a higher index value. The factor of 100 was applied for numerical scaling. The ratio was not intended to represent a biological interaction between GGT and eGFR, and the index was treated as an exploratory derived measure. Because ratio variables may have nontrivial statistical properties and can be susceptible to scale dependence, model misspecification, and mathematical coupling, the composite index was not evaluated on the basis of its association with baPWV alone; its individual components were also examined directly in subsequent analyses. Natural-log-transformed GGT was recalculated directly from the original GGT measurements; the existing log_2_(GGT) variable in the source dataset was not used.

The primary outcome was baPWV analyzed as a continuous variable. As secondary outcomes, elevated baPWV was examined using prespecified cut-points of ≥1400 and ≥1600 cm/s, with ≥1800 cm/s examined in a supplementary analysis (34–36). A baPWV of 1400 cm/s has been used for cardiovascular risk stratification in general health-screening populations, whereas 1600 cm/s has been proposed as a risk-related cut-point in individuals at relatively low cardiovascular risk and 1800 cm/s as a higher-risk cut-point.

### 2.4 Statistical analysis

Baseline characteristics are summarized as mean ± standard deviation, median (interquartile range), or n (%), and compared across index quartiles using one-way analysis of variance, the Kruskal–Wallis test, or the chi-square test as appropriate. The association between the composite index and baPWV was estimated with linear regression, and the association with elevated baPWV with logistic regression, modeling the index per 1-unit increase, per 1-SD increase, and in quartiles (lowest quartile as reference), with a linear trend tested across quartiles. Three nested models were fitted: Model 1 adjusted for age, sex, and BMI; Model 2 added smoking status (never, former, or current), alcohol consumption (none/minimal, light, moderate, heavy, or missing), exercise habit, fatty liver, and corrected ABI; and Model 3 added fasting glucose, triglycerides, HDL-C, LDL-C, uric acid, and systolic and diastolic blood pressure. No missing values were imputed. The 13 participants with missing alcohol-consumption data were represented by a separate category; therefore, the crude model and Models 1–3 each included all 893 participants.

To compare the composite index with its individual components, five models were fitted using the same 893-participant cohort and the full Model 3 covariate set: covariates only; covariates plus ln(GGT); covariates plus eGFR; covariates plus both ln(GGT) and eGFR; and covariates plus the GGT–eGFR composite index. ln(GGT), eGFR, and the composite index were standardized before model entry. For continuous baPWV, model fit was assessed using adjusted R², change in R² relative to the covariates-only model, Akaike information criterion, Bayesian information criterion, and standardized regression coefficients. Partial F tests were used to compare augmented models with the covariates-only model. The incremental statistical value of the derived index was assessed by comparison with models containing its component markers rather than from statistical significance of the index alone, consistent with methodological recommendations for the construction and evaluation of combined or novel biomarkers.

Because the GGT/HDL-C ratio had previously been examined in the same source dataset, an additional exploratory comparison was performed between the GGT–eGFR index and the GGT/HDL-C ratio. To provide a common adjustment set, HDL-C was omitted from both models because it is a component of the GGT/HDL-C ratio. Both indices were standardized before model entry, and model fit was compared using adjusted R², ΔR², AIC, and BIC.

For baPWV ≥1400 and ≥1600 cm/s, model performance was assessed using 10-fold stratified cross-validated out-of-fold predictions. The area under the receiver operating characteristic curve, Brier score, calibration intercept, and calibration slope were calculated using the same cross-validation folds for all models.

Because age and sex are components of the Japanese eGFR equation, sensitivity analyses were performed to examine whether the index–baPWV association depended on this aspect of index construction. The index was standardized separately within men and women, and quartiles were defined separately within each sex. We also recalculated the index using eGFR residualized for age and sex; eGFR was regressed on age and sex, and the residual was added to the overall mean eGFR before recalculation of the index. Sex-stratified models used within-sex standardization and included the Model 3 covariates other than sex. The female model was additionally adjusted for menopausal status. The index-by-sex interaction was examined using both overall and within-sex standardization.

Sensitivity analyses included a complete-case analysis excluding the 13 participants with missing alcohol-consumption data; exclusion of heavy alcohol consumers (>280 g/week); exclusion of participants with fatty liver; exclusion of participants with elevated aminotransferases, defined as AST >40 U/L or ALT >40 U/L; restriction to participants with neither fatty liver nor heavy alcohol use; and exclusion of the 36 participants whose ABI values required format correction. All sensitivity analyses used the fully adjusted Model 3. Multicollinearity was assessed with generalized variance inflation factors. Restricted cubic spline analysis was performed using the fully adjusted Model 3. Three knots were placed at the 10th, 50th, and 90th percentiles of the composite-index distribution (3.053, 4.288, and 6.041, respectively), with the median value (4.288) used as the reference. The model included age, sex, BMI, smoking status, alcohol consumption, exercise, fatty liver, corrected ABI, fasting plasma glucose, triglycerides, HDL-C, LDL-C, uric acid, systolic blood pressure, and diastolic blood pressure. No extreme index values were truncated. The overall association was assessed using a partial F test for the spline terms, and non-linearity was assessed using the F test for the nonlinear spline component. The interaction between GGT and eGFR was examined by entering continuous ln(GGT), continuous eGFR, and their interaction term in the fully adjusted model. ln(GGT) and eGFR were standardized before calculation of the interaction term. The interaction coefficient, 95% confidence interval, and P value were estimated with continuous baPWV as the outcome. As a secondary descriptive analysis, participants were also cross-classified by GGT (below vs at or above the cohort median) and eGFR (≥60 vs <60 mL/min/1.73 m²). Subgroup analyses were considered exploratory and were performed by age, sex, BMI, systolic blood pressure, fasting glucose, triglycerides, HDL-C, uric acid, fatty liver, exercise, smoking, and alcohol use. Subgroup cutoffs were age <52 versus ≥52 years (52 years was the cohort median), BMI <25 versus ≥25 kg/m², SBP <130 versus ≥130 mmHg, FPG <100 versus ≥100 mg/dL, TG <150 versus ≥150 mg/dL, low HDL-C (<40 mg/dL in men or <50 mg/dL in women), and high uric acid (>7 mg/dL in men or >6 mg/dL in women). Smoking was grouped as current versus never/former smoking, and alcohol consumption as any alcohol use versus none/minimal use. Participants with missing alcohol-consumption data were excluded only from the alcohol subgroup analysis. Effect modification was assessed using an interaction term between the standardized composite index and each subgroup variable. Because 12 interaction tests were performed, nominal interaction P values were adjusted using the Benjamini–Hochberg false-discovery-rate procedure. Both nominal and FDR-adjusted interaction P values, together with the sample size in each subgroup, are reported in **Supplementary Table 6**. For the primary analyses, a two-sided P < 0.05 was considered statistically significant. Subgroup analyses were interpreted as exploratory, with emphasis on the interaction tests and their FDR-adjusted P values. Analyses were performed in R software version 4.4.2.

## 3 Results

### 3.1 Baseline characteristics by quartiles of the GGT–eGFR composite index

The 893 participants had a mean age of 51.0 ± 9.5 years, and 584 (65.4%) were men. The four index quartiles contained 223, 222, 224, and 224 participants, respectively. Across ascending index quartiles, participants were older, more often male, and had higher BMI, blood pressure, aminotransferases, fasting glucose, uric acid, total cholesterol, triglycerides, and LDL-C, and lower HDL-C (all P < 0.001; **Table 1**). By construction, GGT rose and eGFR fell across quartiles. The distributions of smoking status and alcohol consumption and the prevalence of fatty liver differed across quartiles, whereas exercise habit did not. Mean baPWV increased monotonically from 1295 cm/s in Q1 to 1531 cm/s in Q4.

**Table 1:** Baseline characteristics of participants according to quartiles of the GGT-eGFR composite index.

Variables	Total (n = 893)	Q1 (n = 223)	Q2 (n = 222)	Q3 (n = 224)	Q4 (n = 224)	P value
**baPWV**	1413.00 ± 242.57	1295.48 ± 160.24	1393.74 ± 206.16	1430.63 ± 216.78	1531.46 ± 303.47	***<0.001***
Sex, n (%)						*<0.001*
Male	584 (65.40)	85 (38.12)	141 (63.51)	165 (73.66)	193 (86.16)	
Female	309 (34.60)	138 (61.88)	81 (36.49)	59 (26.34)	31 (13.84)	
**Age, (years)**	51.03 ± 9.49	47.19 ± 9.58	51.06 ± 9.22	51.35 ± 9.35	54.51 ± 8.39	<0.001
**BMI, (kg/m^2^**)	23.14 ± 3.14	22.14 ± 3.45	22.81 ± 2.74	23.26 ± 3.10	24.35 ± 2.80	***<0.001***
**SBP, (mmHg)**	120.20 ± 14.94	113.14 ± 14.12	119.97 ± 14.44	122.47 ± 14.57	125.17 ± 13.99	***<0.001***
**DBP, (mmHg)**	76.11 ± 9.96	70.71 ± 8.58	75.80 ± 10.12	77.58 ± 9.75	80.31 ± 8.82	***<0.001***
**AST, (IU/L)**	20.84 ± 8.11	17.93 ± 4.71	19.19 ± 5.61	21.56 ± 9.27	24.65 ± 9.87	***<0.001***
**ALT, (IU/L)**	22.71 ± 14.34	16.77 ± 7.14	19.08 ± 8.93	24.23 ± 16.49	30.70 ± 17.48	***<0.001***
**GGT, (IU/L)**	19.00 (14.00, 28.00)	12.00 (10.00, 15.00)	17.00 (14.00, 20.00)	21.00 (17.00, 29.00)	34.00 (24.00, 52.50)	***<0.001***
**FPG, (mg/dl)**	98.03 ± 14.17	93.15 ± 8.63	96.00 ± 9.32	99.08 ± 13.13	103.83 ± 20.19	***<0.001***
**UA, (mg/dl)**	5.26 ± 1.38	4.35 ± 1.15	5.17 ± 1.27	5.38 ± 1.18	6.14 ± 1.28	***<0.001***
**TC, (mg/dl)**	209.77 ± 36.01	202.08 ± 34.61	207.46 ± 35.27	209.51 ± 35.02	219.97 ± 36.95	***<0.001***
**TG, (mg/dl)**	81.00 (54.00, 124.00)	56.00 (40.00, 76.50)	77.00 (51.50, 109.50)	90.00 (60.75, 125.00)	121.00 (74.00, 177.25)	***<0.001***
**HDL-c, (mg/dl)**	53.48 ± 14.55	58.41 ± 14.25	54.22 ± 14.59	51.52 ± 14.64	49.80 ± 13.33	***<0.001***
**LDL-c, (mg/dl)**	127.98 ± 31.79	120.62 ± 29.56	126.83 ± 31.27	129.52 ± 31.81	134.89 ± 32.96	***<0.001***
**eGFR, (mL/min/1.73 m^2^**)	70.40 ± 12.05	82.56 ± 10.87	71.36 ± 7.64	67.47 ± 8.43	60.28 ± 8.59	***<0.001***
**ABI**	1.19 (1.14, 1.23)	1.17 (1.13, 1.22)	1.19 (1.14, 1.22)	1.19 (1.15, 1.24)	1.20 (1.15, 1.25)	***0.001***
Alcohol consumption, n (%)						*<0.001*
None or minimal	566 (63.38)	180 (80.72)	144 (64.86)	138 (61.61)	104 (46.43)	
Light	147 (16.46)	21 (9.42)	39 (17.57)	49 (21.88)	38 (16.96)	
Moderate	90 (10.08)	11 (4.93)	20 (9.01)	20 (8.93)	39 (17.41)	
Heavy	77 (8.62)	7 (3.14)	17 (7.66)	13 (5.80)	40 (17.86)	
Missing	13 (1.46)	4 (1.79)	2 (0.90)	4 (1.79)	3 (1.34)	
Smoking status, n (%)						<0.001
Never	450 (50.39)	146 (65.47)	113 (50.90)	102 (45.54)	89 (39.73)	
Former	248 (27.77)	37 (16.59)	61 (27.48)	67 (29.91)	83 (37.05)	
Current	195 (21.84)	40 (17.94)	48 (21.62)	55 (24.55)	52 (23.21)	
Habit of exercise, n (%)						0.196
No	716 (80.18)	173 (77.58)	171 (77.03)	185 (82.59)	187 (83.48)	
Yes	177 (19.82)	50 (22.42)	51 (22.97)	39 (17.41)	37 (16.52)	
Fatty liver, n (%)						*<0.001*
No	634 (71.00)	188 (84.30)	172 (77.48)	155 (69.20)	119 (53.12)	
Yes	259 (29.00)	35 (15.70)	50 (22.52)	69 (30.80)	105 (46.88)	

Data are presented as mean ± SD, median (IQR), or n (%), as appropriate. The GGT-eGFR composite index was calculated as ln(GGT) × 100/eGFR. P values were calculated using one-way analysis of variance for normally distributed continuous variables, the Kruskal-Wallis test for skewed continuous variables, and chi-square tests for categorical variables. ABI, ankle-brachial index; ALT, alanine aminotransferase; AST, aspartate aminotransferase; baPWV, brachial-ankle pulse wave velocity; BMI, body mass index; DBP, diastolic blood pressure; eGFR, estimated glomerular filtration rate; FPG, fasting plasma glucose; GGT, gamma-glutamyltransferase; HDL-C, high-density lipoprotein cholesterol; LDL-C, low-density lipoprotein cholesterol; SBP, systolic blood pressure; TC, total cholesterol; TG, triglycerides; UA, uric acid.

P - value less than 0.05 is expressed in bold.

### 3.2 Association of the GGT–eGFR composite index with baPWV

In the crude analysis, each 1-unit increase in the composite index was associated with a 67.01 cm/s higher baPWV (95% CI 54.59–79.44; P <0.001). The estimate attenuated after adjustment but remained statistically significant in Model 3: β = 16.91 cm/s per 1-unit increase (95% CI 4.66–29.16; P = 0.007) and β = 20.46 cm/s per 1-SD increase (95% CI 5.64–35.28; P = 0.007) (**Table 2**). Relative to Q1, baPWV was 74.77 cm/s higher in Q4 (95% CI 33.96–115.57; P <0.001). When quartiles were modeled ordinally, each one-quartile increase was associated with a 23.16 cm/s higher baPWV (95% CI 10.33–35.98; P <0.001). No meaningful multicollinearity was detected; the adjusted GVIF, expressed as GVIF^(1/(2×Df)), was 1.32 for the composite index (**Supplementary Table 1**). Univariable associations of the composite index and other covariates with baPWV are presented in **Supplementary Table 2**.

**Table 2:** Association of the GGT-eGFR composite index with baPWV in multivariable linear regression models.

Variable	Crude model (n = 893)		Model 1 (n = 893)		Model 2 (n = 893)		Model 3 (n = 893)	
	β (95%CI)	P value	β (95%CI)	P value	β (95%CI)	P value	β (95%CI)	P value
Composite index, per 1-unit increase	67.01 (54.59, 79.44)	<0.001	33.48 (20.51, 46.45)	<0.001	30.61 (17.42, 43.81)	<0.001	16.91 (4.66, 29.16)	0.007
Composite index, per 1-SD increase	81.07 (66.03, 96.10)	<0.001	40.50 (24.81, 56.19)	<0.001	37.03 (21.07, 53.00)	<0.001	20.46 (5.64, 35.28)	0.007
Composite-index quartile								
Q1	Ref		Ref		Ref		Ref	
Q2	98.26 (55.88, 140.64)	<0.001	42.22 (2.77, 81.66)	0.036	44.19 (5.15, 83.23)	0.027	9.93 (-23.96, 43.82)	0.566
Q3	135.15 (92.87, 177.44)	<0.001	70.78 (30.34, 111.22)	<0.001	69.11 (28.91, 109.31)	<0.001	21.04 (-14.27, 56.35)	0.242
Q4	235.98 (193.70, 278.26)	<0.001	126.96 (83.05, 170.87)	<0.001	119.07 (74.54, 163.61)	<0.001	74.77 (33.96, 115.57)	<0.001
Per one-quartile increase (ordinal)	74.49 (61.11, 87.88)	<0.001	40.80 (26.96, 54.64)	<0.001	37.90 (23.88, 51.91)	<0.001	23.16 (10.33, 35.98)	<0.001

All regression models included 893 participants.

Model 1 adjusted for age, sex, and BMI.

Model 2 additionally adjusted for smoking status (never, former, current), alcohol consumption (none/minimal, light, moderate, heavy), exercise, fatty liver, and corrected ABI.

Model 3 additionally adjusted for FPG, TG, HDL-C, LDL-C, uric acid, SBP, and DBP.

ABI, ankle-brachial index; baPWV, brachial-ankle pulse wave velocity; BMI, body mass index; CI, confidence interval; DBP, diastolic blood pressure; eGFR, estimated glomerular filtration rate; FPG, fasting plasma glucose; GGT, gamma-glutamyltransferase; HDL-C, high-density lipoprotein cholesterol; LDL-C, low-density lipoprotein cholesterol; SBP, systolic blood pressure; SD, standard deviation; TG, triglycerides.

### 3.3 Association with elevated baPWV

The index was also associated with dichotomized arterial stiffness (**Table 3**). In Model 3, each 1-SD increment raised the odds of baPWV ≥ 1400 cm/s by 32% (odds ratio 1.32 (95% CI 1.06–1.64; P = 0.014) and of baPWV ≥ 1600 cm/s by 36% (1.36, 95% CI 1.04–1.79; P = 0.024). Compared with Q1, the fully adjusted odds ratio for the highest quartile was 2.47 (95% CI 1.35–4.53) for ≥ 1400 cm/s and 4.02 (95% CI 1.54–10.49) for ≥ 1600 cm/s, with significant trends (P = 0.003 and 0.006, respectively).

**Table 3:** Association of the GGT-eGFR composite index with elevated baPWV in logistic regression models.

Variable	Crude model (n = 893)		Model 1 (n = 893)		Model 2 (n = 893)		Model 3 (n = 893)	
	OR (95%CI)	P value	OR (95%CI)	P value	OR (95%CI)	P value	OR (95%CI)	P value
baPWV ≥1400 cm/s								
Per 1-SD increase	1.91 (1.64, 2.23)	<0.001	1.54 (1.29, 1.85)	<0.001	1.47 (1.22, 1.77)	<0.001	1.32 (1.06, 1.64)	0.014
Q1	Ref		Ref		Ref		Ref	
Q2	2.45 (1.62, 3.70)	<0.001	1.84 (1.17, 2.90)	0.008	1.86 (1.18, 2.95)	0.008	1.44 (0.86, 2.43)	0.166
Q3	3.16 (2.10, 4.77)	<0.001	2.38 (1.51, 3.78)	<0.001	2.36 (1.48, 3.77)	<0.001	1.68 (0.99, 2.86)	0.055
Q4	6.11 (4.03, 9.26)	<0.001	3.54 (2.16, 5.78)	<0.001	3.20 (1.92, 5.34)	<0.001	2.47 (1.35, 4.53)	0.003
Per one-quartile increase (ordinal)	1.75 (1.54, 1.99)	<0.001	1.48 (1.27, 1.73)	<0.001	1.44 (1.22, 1.68)	<0.001	1.32 (1.10, 1.60)	0.003
baPWV ≥1600 cm/s								
Per 1-SD increase	1.85 (1.56, 2.20)	<0.001	1.59 (1.29, 1.96)	<0.001	1.57 (1.25, 1.95)	<0.001	1.36 (1.04, 1.79)	0.024
Q1	Ref		Ref		Ref		Ref	
Q2	4.15 (1.94, 8.90)	<0.001	3.19 (1.41, 7.21)	0.005	3.31 (1.45, 7.54)	0.004	2.09 (0.84, 5.24)	0.114
Q3	4.11 (1.92, 8.81)	<0.001	2.98 (1.30, 6.83)	0.010	3.06 (1.32, 7.10)	0.009	1.68 (0.65, 4.35)	0.285
Q4	10.81 (5.24, 22.30)	<0.001	6.57 (2.90, 14.93)	<0.001	6.21 (2.69, 14.33)	<0.001	4.02 (1.54, 10.49)	0.004
Per one-quartile increase (ordinal)	1.93 (1.61, 2.31)	<0.001	1.65 (1.33, 2.05)	<0.001	1.60 (1.28, 2.00)	<0.001	1.47 (1.12, 1.94)	0.006

All regression models included 893 participants.

Model 1 adjusted for age, sex, and BMI.

Model 2 additionally adjusted for smoking status (never, former, current), alcohol consumption (none/minimal, light, moderate, heavy), exercise, fatty liver, and corrected ABI.

Model 3 additionally adjusted for FPG, TG, HDL-C, LDL-C, uric acid, SBP, and DBP.

ABI, ankle-brachial index; baPWV, brachial-ankle pulse wave velocity; BMI, body mass index; CI, confidence interval; DBP, diastolic blood pressure; eGFR, estimated glomerular filtration rate; FPG, fasting plasma glucose; GGT, gamma-glutamyltransferase; HDL-C, high-density lipoprotein cholesterol; LDL-C, low-density lipoprotein cholesterol; SBP, systolic blood pressure; SD, standard deviation; TG, triglycerides.

### 3.4 Direct comparison with ln(GGT) and eGFR

Direct model comparisons are shown in **Table 4**. For continuous baPWV, the covariates-only model had an adjusted R² of 0.5000. Adding ln(GGT), eGFR, or both components resulted in ΔR² values of 0.0010, 0.0020, and 0.0032, respectively. The composite-index model had an adjusted R² of 0.5036 and a ΔR² of 0.0041 relative to the covariates-only model (P = 0.007). This model also had the lowest AIC and BIC among the models examined, although the absolute differences were modest. The standardized β coefficient for the composite index was 0.084.

**Table 4:** Direct comparison of the composite index with ln(GGT) and eGFR for continuous baPWV.

Model	n	Adjusted R²	ΔR² vs covariates-only model	AIC	BIC	P for added marker(s)	Standardized β coefficient(s)	Unstandardized β (95% CI), cm/s per 1-SD increase	Marker-specific P value(s)
Covariates only	893	0.5000	0.0000	11742.45	11838.34	—	–	—	—
Covariates + ln(GGT)	893	0.5005	0.0010	11742.56	11843.25	0.175	0.041	ln(GGT): 9.83 (-4.37, 24.03)	ln(GGT): 0.175
Covariates + eGFR	893	0.5015	0.0020	11740.78	11841.47	0.059	-0.054	eGFR: -13.04 (-26.56, 0.47)	eGFR: 0.059
Covariates + ln(GGT) + eGFR	893	0.5021	0.0032	11740.65	11846.13	0.059	ln(GGT): 0.043; eGFR: −0.056	ln(GGT): 10.44 (-3.76, 24.63); eGFR: -13.47 (-26.99, 0.05)	ln(GGT): 0.149; eGFR: 0.051
Covariates + GGT–eGFR composite index	893	0.5036	0.0041	11736.96	11837.65	0.007	0.084	GGT–eGFR index: 20.46 (5.64, 35.28)	GGT–eGFR index: 0.007

All models used the same 893-participant cohort and the full Model 3 covariate set. Missing alcohol-consumption data were modeled as a separate category. ln(GGT), eGFR, and the GGT–eGFR composite index were standardized before model entry; marker coefficients therefore represent the adjusted difference in baPWV per 1-SD increase. Standardized β coefficients were obtained after standardizing baPWV. ΔR² is the change in ordinary R² relative to the covariates-only model. P values for added marker(s) were obtained from partial F tests comparing each augmented model with the covariates-only model. AIC, Akaike information criterion; BIC, Bayesian information criterion; CI, confidence interval; eGFR, estimated glomerular filtration rate; GGT, gamma-glutamyltransferase; SD, standard deviation.

For baPWV ≥1400 and ≥1600 cm/s, cross-validated AUC, Brier score, and calibration measures were generally comparable across the five models (**Supplementary Table 3**). Overall, the composite index provided a modest improvement in model fit for continuous baPWV, whereas differences in categorical model performance were limited.

In the exploratory comparison with the GGT/HDL-C ratio, using the same covariate set for both indices, the GGT–eGFR model had an adjusted R² of 0.5041 compared with 0.5002 for the GGT/HDL-C model; the corresponding ΔR² values were 0.0041 and 0.0002. AIC and BIC were 11735.01 and 11830.90 for the GGT–eGFR model and 11742.07 and 11837.96 for the GGT/HDL-C model, respectively (**Supplementary Table 7**).

### 3.5 Dose–response relationship

**Figure 2:**
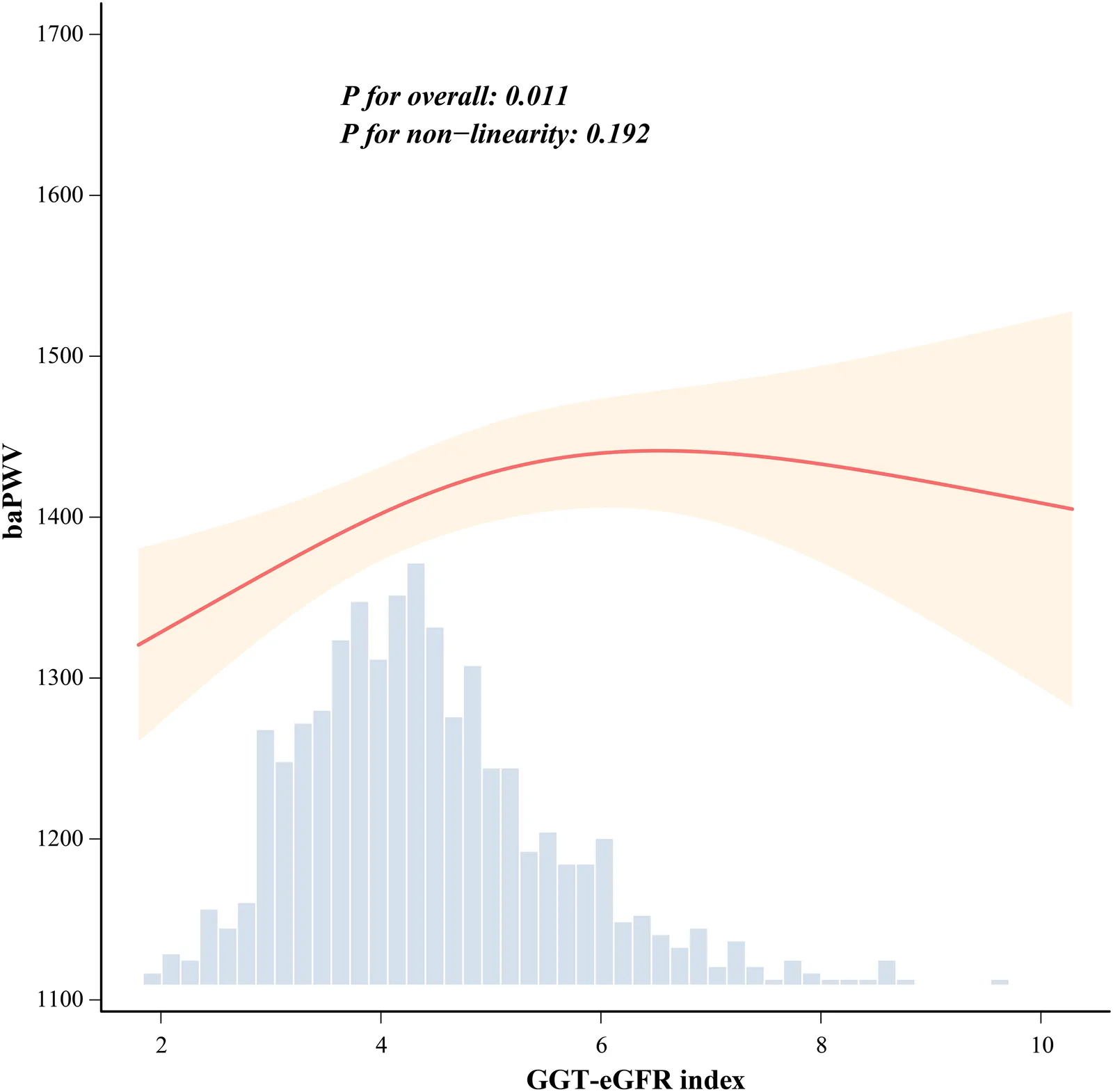
Restricted cubic spline analysis of the association between the GGT–eGFR composite index and adjusted baPWV. Three knots were placed at the 10th, 50th, and 90th percentiles of the index distribution (3.053, 4.288, and 6.041, respectively), with the median value (4.288) as the reference. The model was adjusted for the Model 3 covariates, and no extreme index values were truncated. The solid line represents adjusted baPWV, the shaded area represents the 95% confidence interval, and the histogram shows the distribution of the index. P for overall = 0.011; P for non-linearity = 0.192.

Restricted cubic spline analysis showed an overall association between the composite index and baPWV (P for overall = 0.011). There was no statistically significant evidence of departure from linearity (P for non-linearity = 0.192). An apparent downturn was observed at the upper end of the index distribution, where observations were sparse and the confidence intervals widened (**Figure 2**).

### 3.6 Continuous interaction between GGT and eGFR

In the continuous analysis, there was no statistical evidence of an interaction between ln(GGT) and eGFR. The coefficient for the interaction between standardized ln(GGT) and standardized eGFR was −0.94 cm/s (95% CI −12.21 to 10.32; P = 0.869) in the fully adjusted model (**Supplementary Table 5**).

The original four-group analysis was retained as a secondary descriptive analysis. Compared with participants with low GGT and eGFR ≥60 mL/min/1.73 m², adjusted baPWV was higher by 45.03 cm/s (95% CI 15.67–74.38; P = 0.003) in those with high GGT and preserved eGFR, by 41.05 cm/s (95% CI −4.37–86.47; P = 0.076) in those with low GGT and eGFR <60 mL/min/1.73 m², and by 88.14 cm/s (95% CI 43.44–132.83; P < 0.001) in those with both high GGT and reduced eGFR.

### 3.7 Subgroup analyses

**Figure 3:**
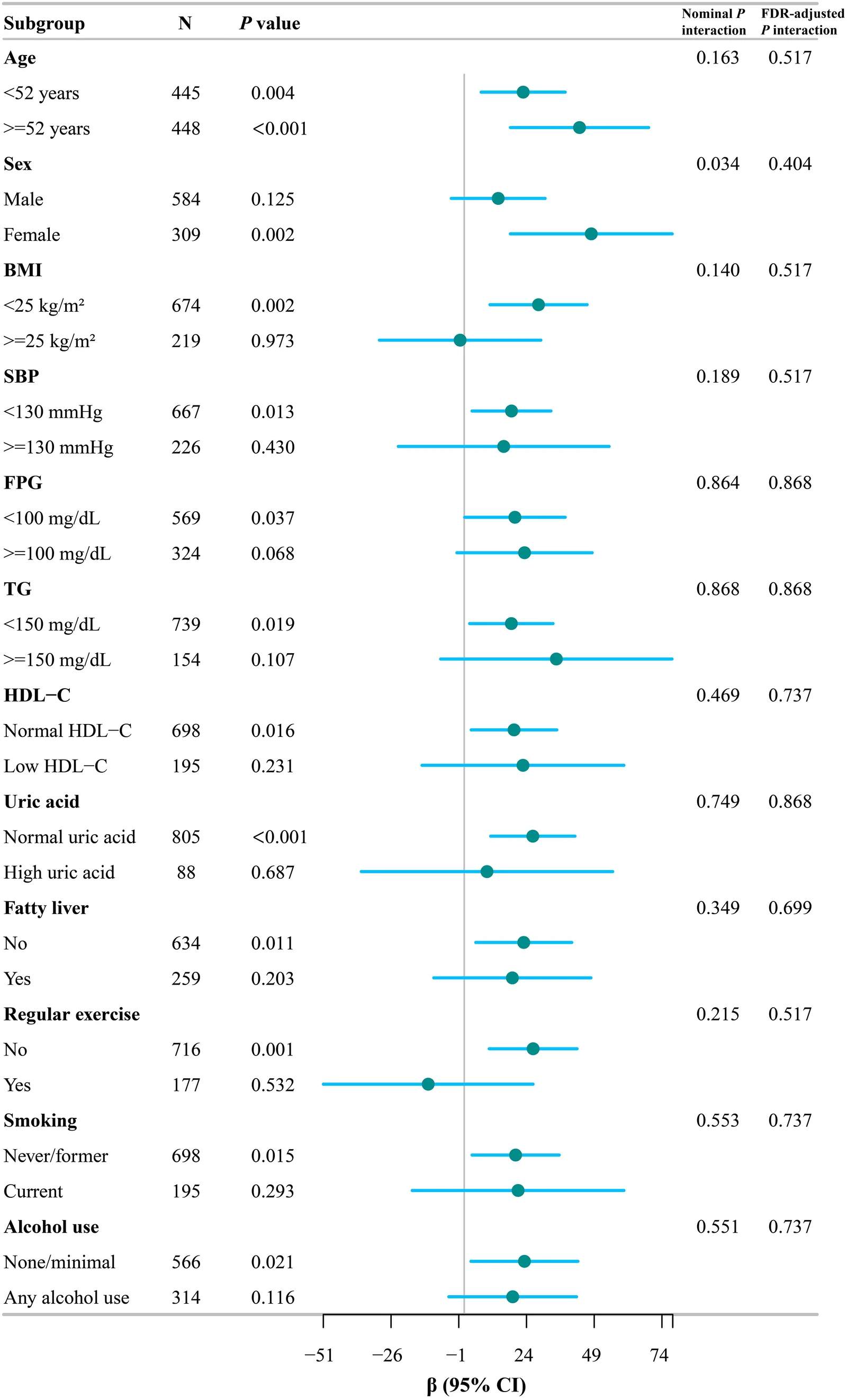
Exploratory subgroup analyses of the association between the GGT–eGFR composite index and baPWV. β coefficients with 95% confidence intervals and subgroup sample sizes are shown. Nominal and Benjamini–Hochberg FDR-adjusted P values are reported for each interaction; detailed results are provided in **Supplementary Table 6**.

Exploratory subgroup analyses are shown in **Figure 3**; **Supplementary Table 6**, with sample sizes reported for each subgroup. Among the 12 interaction tests, sex had a nominal interaction P value of 0.034, but this association did not remain statistically significant after FDR correction (adjusted P = 0.404). No subgroup interaction remained statistically significant after correction for multiple testing.

### 3.8 Sensitivity analyses for the primary association

The association was similar in the complete-case analysis excluding the 13 participants with missing alcohol-consumption data (n = 880; β per 1 SD = 20.75 cm/s, 95% CI 5.83–35.66; P = 0.006) (**Table 5**). After excluding heavy alcohol consumers, the estimate was 20.13 cm/s (n = 816; 95% CI 4.20–36.05; P = 0.013). Corresponding estimates were 22.99 cm/s after excluding participants with fatty liver (n = 634; 95% CI 5.28–40.71; P = 0.011) and 24.16 cm/s after excluding those with AST >40 U/L or ALT >40 U/L (n = 807; 95% CI 7.53–40.80; P = 0.004). Restricting the analysis to participants with neither fatty liver nor heavy alcohol use yielded an estimate of 23.44 cm/s (n = 578; 95% CI 4.22–42.67; P = 0.017). The estimate was also similar after excluding the 36 participants whose ABI values required format correction (n = 857; β = 17.33 cm/s, 95% CI 2.17–32.50; P = 0.025).

**Table 5:** Sensitivity analyses of the association between the GGT-eGFR composite index and baPWV.

Sensitivity analysis	n	β per 1 SD (95% CI), cm/s	P value
Primary analysis retaining missing alcohol as a separate category	893	20.46 (5.64, 35.28)	0.007
Complete-case analysis for alcohol consumption	880	20.75 (5.83, 35.66)	0.006
Excluding heavy alcohol consumers (>280 g/week)	816	20.13 (4.20, 36.05)	0.013
Excluding participants with fatty liver	634	22.99 (5.28, 40.71)	0.011
Excluding participants with elevated AST or ALT (>40 U/L)	807	24.16 (7.53, 40.80)	0.004
Restricting to participants without fatty liver and without heavy alcohol use	578	23.44 (4.22, 42.67)	0.017
Excluding all records whose ABI required format correction	857	17.33 (2.17, 32.50)	0.025

Values are fully adjusted Model 3 β coefficients with 95% CIs and represent the change in baPWV per 1-SD increase in the composite index. The SD was fixed to that calculated in the 893-participant primary cohort. The primary analysis retained the 13 participants with missing alcohol-consumption data as a separate category; the complete-case analysis excluded those 13 participants. Heavy alcohol consumption was defined as >280 g/week. Elevated liver enzymes were operationally defined as AST >40 U/L or ALT >40 U/L. The ABI sensitivity analysis excluded all 36 participants whose ABI required decimal-place correction. ABI, ankle-brachial index; ALT, alanine aminotransferase; AST, aspartate aminotransferase; baPWV, brachial-ankle pulse wave velocity; CI, confidence interval; SD, standard deviation.

The main association was also similar when the index was standardized separately within men and women (β per 1 SD = 21.15 cm/s, 95% CI 7.31–34.99; P = 0.003) and when eGFR was residualized for age and sex before recalculation of the index (β = 15.37 cm/s, 95% CI 1.53–29.20; P = 0.030) (**Supplementary Table 4**). Sex-specific quartiles showed a similar graded pattern, with an adjusted difference of 56.56 cm/s for Q4 versus Q1 (95% CI 19.23–93.89; P = 0.003). In sex-stratified analyses using within-sex standardization, the estimates were 13.67 cm/s (95% CI −3.78–31.11; P = 0.125) in men and 36.68 cm/s (95% CI 13.82–59.54; P = 0.002) in women. Additional adjustment for menopausal status changed the estimate in women little (36.38 cm/s, 95% CI 13.50–59.27; P = 0.002). The index-by-sex interaction was attenuated after within-sex standardization (P for interaction = 0.090).

## 4 Discussion

In this cross-sectional analysis of 893 Japanese adults, a higher GGT–eGFR composite index remained associated with higher baPWV after multivariable adjustment. Each 1-SD increase in the index was associated with a 20.46 cm/s higher baPWV, and baPWV was 74.77 cm/s higher in Q4 than in Q1. Associations were also observed for baPWV thresholds of 1400 and 1600 cm/s, and the primary association was generally similar across the sensitivity analyses.

The direct model comparisons provide a more qualified assessment of the derived index. This comparison is particularly important for a ratio-derived index, because an association of the derived variable alone does not establish that it contains information beyond its constituent variables (37–41). For continuous baPWV, the composite-index model produced the largest increase in explained variance and the lowest information criteria among the models examined, although the magnitude of improvement was modest. The index therefore appears to summarize the opposing contributions of GGT and eGFR in a single model term. For categorical baPWV outcomes, discrimination and calibration were broadly comparable across models. Overall, the statistical differences between the composite index and models containing its individual components were modest. The index should therefore be regarded as an exploratory derived measure rather than an established predictor or a replacement for GGT and eGFR considered separately.

These results are consistent with, and extend, prior work on the individual components. Serum GGT has been related to baPWV in several East Asian populations, and elevated GGT tracks metabolic syndrome, fatty liver, and cardiovascular mortality (14–20); reduced eGFR is likewise associated with stiffer arteries and adverse renal and cardiovascular outcomes across cohorts spanning the general population to established chronic kidney disease (23, 25, 26, 42). In the same Japanese dataset used here, separate secondary analyses have linked baPWV to eGFR, the triglyceride-glucose index, the triglyceride-to-HDL-C ratio, the AST/ALT ratio, and the GGT/HDL-C ratio (26–30), reflecting sustained interest in deriving simple composite markers of vascular ageing from routine panels. The GGT–eGFR index and the previously studied GGT/HDL-C ratio are related but are not interchangeable. Both incorporate GGT, whereas their second components reflect different physiological domains: HDL-C primarily represents lipid metabolism, while eGFR reflects kidney function. In the exploratory common-covariate comparison, the GGT–eGFR model showed somewhat better fit for continuous baPWV than the GGT/HDL-C model. However, the absolute differences were small, and the previous GGT/HDL-C study reported a nonlinear association with baPWV. These findings do not establish superiority of one index over the other, but suggest that they may summarize different biological information. The present study is the first to combine an elevated GGT with a reduced eGFR into a single index and to show that this combination is associated with arterial stiffness; the secondary four-group analysis showed the highest adjusted baPWV among participants with both higher GGT and reduced eGFR. However, the continuous analysis provided no statistical evidence of interaction between ln(GGT) and eGFR.

Several mechanisms plausibly connect the index to arterial stiffening, and they converge on oxidative stress, inflammation, and extracellular-matrix remodeling. GGT participates in the extracellular catabolism of glutathione and, in the presence of iron, generates reactive oxygen species that oxidize low-density lipoprotein; catalytically active GGT is present within atherosclerotic plaques, and higher circulating GGT accompanies subclinical inflammation (8–12). Reactive oxygen species reduce nitric oxide bioavailability, promote endothelial dysfunction, activate matrix metalloproteinases that fragment elastin and increase stiffer collagen, and favor vascular calcification—each a recognized determinant of arterial stiffness (43–49). Declining renal function amplifies the same pathways through fluid and mineral dysregulation, chronic low-grade inflammation, accumulation of uremic solutes, and medial calcification, which together accelerate large-artery stiffening (21–24). These overlapping pathways provide a possible biological context for the observed association, but the present data cannot determine whether the composite index reflects a distinct biological process. The association remained evident after excluding heavy alcohol consumers, participants with fatty liver, and those with elevated aminotransferases, and when the analysis was restricted to participants with neither fatty liver nor heavy alcohol use. Thus, the observed association was not confined to participants with heavy alcohol exposure or overt liver abnormalities, although residual hepatic and lifestyle-related confounding cannot be excluded.

Two further observations deserve comment. First, the sex-specific findings should not be regarded as established effect modification. Although the estimated association was larger in women, the nominal interaction by sex did not remain statistically significant after correction for multiple testing (FDR-adjusted P = 0.404). The interaction was also attenuated after the index was standardized separately within each sex (P for interaction = 0.090). Together, these analyses indicate that the apparent sex difference is sensitive to both multiple-testing correction and index scaling and should therefore be considered exploratory. Second, the spline analysis showed no statistically significant evidence of departure from linearity. The fitted curve turned downward at the upper end of the index distribution, where observations were sparse and confidence intervals were wide. The present analysis therefore does not establish a strictly linear association or exclude the possibility of a threshold.

The index can be calculated from routinely measured GGT and creatinine-based eGFR without additional laboratory testing. However, the present cross-sectional analysis does not establish its value for clinical screening, risk prediction, or treatment guidance. These potential applications require evaluation in prospective and externally validated studies.

This study has several strengths, including a well-characterized health check-up sample, a standardized and reproducible measure of arterial stiffness, extensive adjustment for demographic, lifestyle, metabolic, and hemodynamic covariates, and triangulation of the primary finding with spline modeling, joint stratification, subgroup testing, and multiple sensitivity analyses. Limitations should also be acknowledged. The cross-sectional design precludes causal inference and cannot establish the temporal ordering of a high index and arterial stiffening. The data derive from a single Japanese check-up center, so generalization to other ethnic and clinical groups requires confirmation. GGT and eGFR were each measured once, and the index cannot capture longitudinal change. eGFR was estimated from creatinine rather than measured directly, and cystatin C–based estimates were unavailable. Information on medication was used for exclusion, but detailed data on diet and physical-activity intensity were limited; menopausal status was questionnaire-based, and circulating sex hormone measurements were unavailable. Finally, the weakening of the estimate with full adjustment indicates that part of the association is shared with conventional metabolic and blood-pressure factors.

Future studies should examine the longitudinal association of the GGT–eGFR index with changes in arterial stiffness and cardiovascular outcomes, compare its performance with established risk measures, and assess its reproducibility in independent populations. Prospective and external validation will be necessary before any clinical application of the index can be considered.

## 5 Conclusion

In this cross-sectional secondary analysis of a Japanese health check-up population, a higher exploratory GGT–eGFR-derived index remained associated with higher baPWV after multivariable adjustment. Direct comparisons with ln(GGT) and eGFR showed only modest differences in model fit, and prospective external validation is required before clinical application can be considered.

## Data Availability

The original contributions presented in the study are included in the article/**Supplementary Material**. Further inquiries can be directed to the corresponding author.
