# Supplementary material for: The gamma-glutamyltransferase-to-estimated glomerular filtration rate composite index is associated with arterial stiffness: a cross-sectional secondary analysis of a health check-up population
Source: Front Endocrinol (Lausanne). 2026 Aug 28;17:1930792. doi: 10.3389/fendo.2026.1930792 (PMC13561847; doi:10.3389/fendo.2026.1930792)
Supplement: Supplementary file 1 [file Table1.docx]

Supplementary Material

**Table S1.** Variance inflation factors for the fully adjusted model.

**Table S2.** Univariable linear regression analyses of factors associated with baPWV.

**Table S3.** Comparison of categorical-outcome model performance.

**Table S4.** Sensitivity analyses addressing index construction, mathematical coupling, and sex.

**Table S5.** Continuous interaction between ln(GGT) and eGFR and secondary four-group analysis.

**Table S6.** Detailed exploratory subgroup analyses.

**Table S7.** Exploratory comparison of the GGT–eGFR-derived index with the GGT/HDL-C ratio.

| **Table S1.** Variance inflation factors for the fully adjusted model | | | |
| --- | --- | --- | --- |
| Model term | GVIF | Df | GVIF^(1/(2×Df)) |
| Sex | 2.3454 | 1 | 1.5315 |
| Age | 1.2981 | 1 | 1.1394 |
| BMI | 1.6653 | 1 | 1.2905 |
| Smoking status | 1.5618 | 2 | 1.1179 |
| Alcohol consumption | 1.5485 | 4 | 1.0562 |
| Regular exercise | 1.0797 | 1 | 1.0391 |
| Fatty liver | 1.6103 | 1 | 1.2690 |
| ABI | 1.1077 | 1 | 1.0525 |
| FPG | 1.2394 | 1 | 1.1133 |
| TG | 1.4737 | 1 | 1.2140 |
| HDL-C | 1.5755 | 1 | 1.2552 |
| LDL-C | 1.1596 | 1 | 1.0768 |
| Uric acid | 1.9749 | 1 | 1.4053 |
| SBP | 5.4595 | 1 | 2.3366 |
| DBP | 5.7796 | 1 | 2.4041 |
| GGT–eGFR composite index | 1.7415 | 1 | 1.3197 |
| Generalized variance inflation factors were calculated in the 893-participant fully adjusted Model 3 containing the standardized composite index. Smoking status had 2 degrees of freedom and alcohol consumption had 4 degrees of freedom because missing alcohol data were modeled as a separate category. GVIF^(1/(2×Df)) is reported to permit comparison across terms with different degrees of freedom; values >5 were considered suggestive of potentially important multicollinearity. Df, degrees of freedom; GVIF, generalized variance inflation factor. | | | |
|  |  |  |  |
|  |  |  |  |
|  |  |  |  |
|  |  |  |  |
|  |  |  |  |
|  |  |  |  |
|  |  |  |  |
|  |  |  |  |

| **Table S2.** Univariable linear regression analyses of factors associated with baPWV. | | | |
| --- | --- | --- | --- |
| **Variable** | n | **Unadjusted β (95% CI), cm/s** | **P value** |
| **Composite index, per 1 SD** | 893 | 81.07 (66.03, 96.10) | ***<0.001*** |
| **Female vs male** | 893 | -54.15 (-87.47, -20.83) | ***0.001*** |
| **Age, per year** | 893 | 12.65 (11.20, 14.11) | ***<0.001*** |
| **BMI, per kg/m²** | 893 | 5.04 (-0.04, 10.11) | 0.052 |
| **SBP, per mmHg** | 893 | 8.46 (7.55, 9.37) | ***<0.001*** |
| **DBP, per mmHg** | 893 | 11.43 (10.01, 12.84) | ***<0.001*** |
| **AST, per U/L** | 893 | 3.28 (1.32, 5.23) | ***0.001*** |
| **ALT, per U/L** | 893 | 1.54 (0.43, 2.64) | ***0.007*** |
| **ln(GGT), per 1 SD** | 893 | 48.02 (32.39, 63.66) | ***<0.001*** |
| **FPG, per mg/dL** | 893 | 4.22 (3.13, 5.31) | ***<0.001*** |
| **Uric acid, per mg/dL** | 893 | 22.23 (10.74, 33.73) | ***<0.001*** |
| **Total cholesterol, per mg/dL** | 893 | 0.74 (0.30, 1.18) | ***<0.001*** |
| **Triglycerides, per mg/dL** | 893 | 0.44 (0.23, 0.65) | ***<0.001*** |
| **HDL-C, per mg/dL** | 893 | -1.25 (-2.35, -0.16) | ***0.025*** |
| **LDL-C, per mg/dL** | 893 | 0.66 (0.16, 1.16) | ***0.009*** |
| **eGFR, per 1 SD** | 893 | -75.47 (-90.63, -60.31) | ***<0.001*** |
| **ABI, per unit** | 893 | 256.89 (87.21, 426.57) | ***0.003*** |
| **Former vs never smoking** | 893 | 37.30 (-0.31, 74.91) | 0.052 |
| **Current vs never smoking** | 893 | 14.90 (-25.87, 55.68) | 0.473 |
| **Light vs none/minimal alcohol** | 893 | -3.72 (-47.80, 40.36) | 0.868 |
| **Moderate vs none/minimal alcohol** | 893 | 16.44 (-37.60, 70.47) | 0.551 |
| **Heavy vs none/minimal alcohol** | 893 | 52.97 (-4.86, 110.81) | 0.073 |
| **Missing vs none/minimal alcohol** | 893 | -12.50 (-146.06, 121.07) | 0.854 |
| **Regular exercise: yes vs no** | 893 | 17.06 (-22.91, 57.03) | 0.403 |
| **Fatty liver: yes vs no** | 893 | 94.70 (60.13, 129.27) | ***<0.001*** |
| Values are univariable β coefficients with 95% CIs. Continuous-variable coefficients represent the change in baPWV for the stated unit; ln(GGT), eGFR, and the composite index are reported per 1-SD increase where indicated. Natural-log-transformed GGT was recalculated directly from the original GGT values; the source log₂(GGT) variable was not used. Smoking status was modeled using mutually exclusive never, former, and current categories, with never smoking as the reference. Alcohol consumption was modeled as a five-level categorical variable, with none/minimal consumption as the reference and missing alcohol data retained as a separate category. ABI, ankle-brachial index; ALT, alanine aminotransferase; AST, aspartate aminotransferase; baPWV, brachial-ankle pulse wave velocity; BMI, body mass index; CI, confidence interval; DBP, diastolic blood pressure; eGFR, estimated glomerular filtration rate; FPG, fasting plasma glucose; GGT, gamma-glutamyltransferase; HDL-C, high-density lipoprotein cholesterol; LDL-C, low-density lipoprotein cholesterol; SBP, systolic blood pressure; SD, standard deviation; TC, total cholesterol; TG, triglycerides. | | | |
|  |  |  |  |
|  |  |  |  |
|  |  |  |  |
|  |  |  |  |
|  |  |  |  |
|  |  |  |  |
|  |  |  |  |
|  |  |  |  |
|  |  |  |  |
|  |  |  |  |

| **Table S3.** Comparison of categorical-outcome model performance | | | | | | | | |
| --- | --- | --- | --- | --- | --- | --- | --- | --- |
| **Outcome** | **Model** | **n** | **Events** | **10-fold CV AUC** | **ΔAUC** | **Brier score** | **Calibration intercept** | **Calibration slope** |
| **baPWV ≥1400 cm/s** | Covariates only | 893 | 392 | 0.8282 | 0.0000 | 0.1688 | -0.0217 | 0.8892 |
|  | Covariates + ln(GGT) | 893 | 392 | 0.8277 | -0.0005 | 0.1689 | -0.0252 | 0.8788 |
|  | Covariates + eGFR | 893 | 392 | 0.8277 | -0.0005 | 0.1693 | -0.0242 | 0.8794 |
|  | Covariates + ln(GGT) + eGFR | 893 | 392 | 0.8270 | -0.0012 | 0.1693 | -0.0277 | 0.8702 |
|  | Covariates + GGT–eGFR composite index | 893 | 392 | 0.8291 | 0.0009 | 0.1681 | -0.0246 | 0.8819 |
| **baPWV ≥1600 cm/s** | Covariates only | 893 | 145 | 0.8868 | 0.0000 | 0.0920 | -0.1572 | 0.8509 |
|  | Covariates + ln(GGT) | 893 | 145 | 0.8857 | -0.0010 | 0.0923 | -0.1649 | 0.8451 |
|  | Covariates + eGFR | 893 | 145 | 0.8890 | 0.0022 | 0.0909 | -0.1677 | 0.8354 |
|  | Covariates + ln(GGT) + eGFR | 893 | 145 | 0.8878 | 0.0010 | 0.0912 | -0.1752 | 0.8297 |
|  | Covariates + GGT–eGFR composite index | 893 | 145 | 0.8882 | 0.0015 | 0.0909 | -0.1621 | 0.8441 |
| All models used the same 893-participant cohort and the full Model 3 covariate set; missing alcohol-consumption data were modeled as a separate category. AUC, Brier score, calibration intercept, and calibration slope were calculated from 10-fold stratified cross-validated out-of-fold predictions using a fixed random seed. ΔAUC was calculated relative to the covariates-only model. Values closer to 0 for the calibration intercept and closer to 1 for the calibration slope indicate better calibration. AUC, area under the receiver operating characteristic curve; baPWV, brachial-ankle pulse wave velocity; CV, cross-validation. | | | | | | | | |
|  |  |  |  |  |  |  |  |  |
|  |  |  |  |  |  |  |  |  |
|  |  |  |  |  |  |  |  |  |

| **Table S4.** Sensitivity analyses addressing index construction, mathematical coupling, and sex | | | |
| --- | --- | --- | --- |
| **Analysis** | **n** | **β (95% CI), cm/s** | **P value** |
| **Index-construction sensitivity** |  |  |  |
| Primary index, overall standardization | 893 | 20.46 (5.64, 35.28) | 0.007 |
| Sex-specific standardization | 893 | 21.15 (7.31, 34.99) | 0.003 |
| Alternative index using age/sex-residualized eGFR | 893 | 15.37 (1.53, 29.20) | 0.030 |
| Sex-specific quartiles: Q2 vs Q1 | 893 | 17.54 (-15.18, 50.26) | 0.293 |
| Sex-specific quartiles: Q3 vs Q1 | 893 | 46.94 (12.52, 81.36) | 0.008 |
| Sex-specific quartiles: Q4 vs Q1 | 893 | 56.56 (19.23, 93.89) | 0.003 |
| **Sex-specific analyses** |  |  |  |
| Male | 584 | 13.67 (-3.78, 31.11) | 0.125 |
| Female | 309 | 36.68 (13.82, 59.54) | 0.002 |
| Female, additionally adjusted for menopausal status | 309 | 36.38 (13.50, 59.27) | 0.002 |
| **Sex-interaction sensitivity** |  |  |  |
| Overall-standardized index × sex | 893 | 31.49 (2.43, 60.55) | 0.034 |
| Sex-specific-standardized index × sex | 893 | 20.74 (-3.25, 44.72) | 0.090 |
| All analyses used the 893-participant fully adjusted Model 3, with missing alcohol-consumption data modeled as a separate category. Sex-specific standardization was performed separately within men and women. Sex-specific quartiles were defined separately within each sex and then pooled. For the alternative index, eGFR was regressed on age and sex; the residual was added to the overall mean eGFR to preserve the original scale before recalculating ln(GGT) × 100/eGFR. Sex-stratified analyses omitted sex from the covariate set and used within-sex standardization. The female sensitivity model additionally adjusted for menopausal status. Quartile coefficients are adjusted differences relative to Q1; all other coefficients are per 1-SD increase or interaction-term coefficients, as indicated. CI, confidence interval; eGFR, estimated glomerular filtration rate; GGT, gamma-glutamyltransferase; SD, standard deviation. | | | |
|  |  |  |  |
|  |  |  |  |
|  |  |  |  |
|  |  |  |  |
|  |  |  |  |
|  |  |  |  |
|  |  |  |  |
|  |  |  |  |
|  |  |  |  |

| **Table S5.** Continuous interaction between ln(GGT) and eGFR and secondary four-group analysis | | | |
| --- | --- | --- | --- |
| **Analysis/group** | **n** | **β (95% CI), cm/s** | **P value** |
| **Continuous ln(GGT) × eGFR interaction** |  |  |  |
| Mean-centered ln(GGT) × mean-centered eGFR | 893 | -0.13 (-1.72, 1.46) | 0.869 |
| Standardized ln(GGT) × standardized eGFR | 893 | -0.94 (-12.21, 10.32) | 0.869 |
| **Secondary four-group analysis** |  |  |  |
| Low GGT + eGFR ≥60 | 374 | Reference |  |
| High GGT + eGFR ≥60 | 351 | 45.03 (15.67, 74.38) | 0.003 |
| Low GGT + eGFR <60 | 69 | 41.05 (-4.37, 86.47) | 0.076 |
| High GGT + eGFR <60 | 99 | 88.14 (43.44, 132.83) | <0.001 |
| Binary high-GGT × reduced-eGFR interaction | 893 | 2.06 (-56.50, 60.62) | 0.945 |
| The continuous interaction models included continuous ln(GGT), continuous eGFR, and their product term in the same 893-participant fully adjusted Model 3; missing alcohol-consumption data were modeled as a separate category. The reported coefficient and P value refer to the interaction term. In the mean-centered analysis, ln(GGT) and eGFR were centered before their product was calculated; in the standardized analysis, both variables were standardized first. Because baPWV is continuous, these are statistical interaction terms and should not be described as multiplicative interactions. A non-significant interaction does not demonstrate that the component effects are additive. The four-group analysis is secondary and descriptive. High GGT was defined as GGT ≥19 U/L (the cohort median), and reduced eGFR as <60 mL/min/1.73 m². CI, confidence interval; eGFR, estimated glomerular filtration rate; GGT, gamma-glutamyltransferase. | | | |
|  |  |  |  |
|  |  |  |  |
|  |  |  |  |
|  |  |  |  |
|  |  |  |  |
|  |  |  |  |
|  |  |  |  |
|  |  |  |  |
|  |  |  |  |

| **Table S6.** Detailed exploratory subgroup analyses | | | | | | | |
| --- | --- | --- | --- | --- | --- | --- | --- |
| **Subgroup** | **n** | **β per 1 SD** | **Lower 95% CI** | **Upper 95% CI** | **P value** | **Nominal P for interaction** | **FDR-adjusted P for interaction** |
| Age |  |  |  |  |  | 0.163 | 0.517 |
| <52 years | 445 | 22.78 | 7.26 | 38.30 | 0.004 |  |  |
| ≥52 years | 448 | 43.58 | 18.06 | 69.10 | <0.001 |  |  |
| Sex |  |  |  |  |  | 0.034 | 0.404 |
| Male | 584 | 13.56 | -3.75 | 30.87 | 0.125 |  |  |
| Female | 309 | 47.92 | 18.05 | 77.79 | 0.002 |  |  |
| BMI |  |  |  |  |  | 0.140 | 0.517 |
| <25 kg/m² | 674 | 28.44 | 10.52 | 46.37 | 0.002 |  |  |
| ≥25 kg/m² | 219 | -0.50 | -30.31 | 29.30 | 0.973 |  |  |
| SBP |  |  |  |  |  | 0.189 | 0.517 |
| <130 mmHg | 667 | 18.46 | 3.90 | 33.02 | 0.013 |  |  |
| ≥130 mmHg | 226 | 15.59 | -23.31 | 54.50 | 0.430 |  |  |
| FPG |  |  |  |  |  | 0.864 | 0.868 |
| <100 mg/dL | 569 | 19.73 | 1.16 | 38.30 | 0.037 |  |  |
| ≥100 mg/dL | 324 | 23.28 | -1.69 | 48.24 | 0.068 |  |  |
| TG |  |  |  |  |  | 0.868 | 0.868 |
| <150 mg/dL | 739 | 18.41 | 3.02 | 33.79 | 0.019 |  |  |
| ≥150 mg/dL | 154 | 35.02 | -7.68 | 77.71 | 0.107 |  |  |
| HDL-C |  |  |  |  |  | 0.469 | 0.737 |
| Normal HDL-C | 698 | 19.40 | 3.59 | 35.20 | 0.016 |  |  |
| Low HDL-C | 195 | 22.70 | -14.56 | 59.95 | 0.231 |  |  |
| Uric acid |  |  |  |  |  | 0.749 | 0.868 |
| Normal uric acid | 805 | 26.34 | 10.77 | 41.91 | <0.001 |  |  |
| High uric acid | 88 | 9.41 | -37.00 | 55.82 | 0.687 |  |  |
| Fatty liver |  |  |  |  |  | 0.349 | 0.699 |
| No | 634 | 22.99 | 5.28 | 40.71 | 0.011 |  |  |
| Yes | 259 | 18.79 | -10.22 | 47.79 | 0.203 |  |  |
| Regular exercise |  |  |  |  |  | 0.215 | 0.517 |
| No | 716 | 26.45 | 10.23 | 42.67 | 0.001 |  |  |
| Yes | 177 | -12.28 | -50.98 | 26.41 | 0.532 |  |  |
| Smoking |  |  |  |  |  | 0.553 | 0.737 |
| Never/former | 698 | 19.98 | 3.89 | 36.07 | 0.015 |  |  |
| Current | 195 | 20.89 | -18.19 | 59.96 | 0.293 |  |  |
| Alcohol use |  |  |  |  |  | 0.551 | 0.737 |
| None/minimal | 566 | 23.25 | 3.51 | 42.99 | 0.021 |  |  |
| Any alcohol use | 314 | 18.91 | -4.68 | 42.49 | 0.116 |  |  |
| β coefficients represent the adjusted difference in baPWV per 1-SD increase in the composite index. Subgroup analyses were exploratory, and the variable used to define a subgroup was omitted from the corresponding subgroup-specific model. Cutoffs were age 52 years, BMI 25 kg/m², SBP 130 mmHg, FPG 100 mg/dL, TG 150 mg/dL, low HDL-C <40 mg/dL in men or <50 mg/dL in women, and high uric acid >7 mg/dL in men or >6 mg/dL in women. Smoking was grouped as current versus never/former, and alcohol use as any use versus none/minimal. Alcohol subgroup analyses used the 880 participants with observed alcohol data; all other subgroup analyses used the 893-participant cohort. Nominal interaction P values were obtained from product terms between the continuous standardized index and subgroup indicator. Benjamini–Hochberg false-discovery-rate correction was applied across all 12 interaction tests. Statistical significance within one subgroup but not another should not be interpreted as evidence of effect modification without a significant interaction test. BMI, body mass index; CI, confidence interval; FDR, false discovery rate; FPG, fasting plasma glucose; HDL-C, high-density lipoprotein cholesterol; SBP, systolic blood pressure; SD, standard deviation; TG, triglycerides. | | | | | | | |
|  |  |  |  |  |  |  |  |
|  |  |  |  |  |  |  |  |
|  |  |  |  |  |  |  |  |
|  |  |  |  |  |  |  |  |
|  |  |  |  |  |  |  |  |
|  |  |  |  |  |  |  |  |
|  |  |  |  |  |  |  |  |

| **Table S7.** Exploratory comparison of the GGT–eGFR-derived index with the GGT/HDL-C ratio | | | | | | | |
| --- | --- | --- | --- | --- | --- | --- | --- |
| **Model** | **n** | **Adjusted R²** | **ΔR² vs common covariates** | **AIC** | **BIC** | **Marker β (95% CI), cm/s per 1 SD** | **P value** |
| Common covariates only | 893 | 0.5006 | 0.0000 | 11740.46 | 11831.56 | — | — |
| Common covariates + GGT–eGFR composite index | 893 | 0.5041 | 0.0041 | 11735.01 | 11830.90 | 20.41 (5.60, 35.22) | 0.007 |
| Common covariates + GGT/HDL-C ratio | 893 | 0.5002 | 0.0002 | 11742.07 | 11837.96 | -4.10 (-16.98, 8.79) | 0.533 |
| All models used the 893-participant cohort and a common covariate set consisting of age, sex, BMI, smoking status, alcohol consumption (including a separate missing-data category), regular exercise, fatty liver, corrected ABI, FPG, TG, LDL-C, uric acid, SBP, and DBP. HDL-C was intentionally omitted from the common covariate set because it is a component of the GGT/HDL-C ratio. Both candidate indices were standardized. ΔR² was calculated relative to the common-covariates-only model. Lower AIC/BIC and higher adjusted R² indicate better statistical fit, but this exploratory comparison does not establish clinical superiority. ABI, ankle-brachial index; AIC, Akaike information criterion; BIC, Bayesian information criterion; BMI, body mass index; CI, confidence interval; DBP, diastolic blood pressure; FPG, fasting plasma glucose; GGT, gamma-glutamyltransferase; HDL-C, high-density lipoprotein cholesterol; LDL-C, low-density lipoprotein cholesterol; SBP, systolic blood pressure; SD, standard deviation; TG, triglycerides. | | | | | | | |
|  |  |  |  |  |  |  |  |
|  |  |  |  |  |  |  |  |
|  |  |  |  |  |  |  |  |
|  |  |  |  |  |  |  |  |
|  |  |  |  |  |  |  |  |
|  |  |  |  |  |  |  |  |
